# Spatial Distribution Analysis of Novel Texture Feature Descriptors for Accurate Breast Density Classification

**DOI:** 10.3390/s22072672

**Published:** 2022-03-30

**Authors:** Haipeng Li, Ramakrishnan Mukundan, Shelley Boyd

**Affiliations:** 1 Department of Computer Science and Software Engineering, University of Canterbury, Christchurch 8140, New Zealand; mukundan@canterbury.ac.nz; 2 Canterbury Breastcare, St. George’s Medical Centre, Christchurch 8014, New Zealand; shelley.boyd@pacificradiology.com

**Keywords:** breast density classification, mammography, local quinary patterns, spatial distribution analysis, texture features

## Abstract

Breast density has been recognised as an important biomarker that indicates the risk of developing breast cancer. Accurate classification of breast density plays a crucial role in developing a computer-aided detection (CADe) system for mammogram interpretation. This paper proposes a novel texture descriptor, namely, rotation invariant uniform local quinary patterns (RIU4-LQP), to describe texture patterns in mammograms and to improve the robustness of image features. In conventional processing schemes, image features are obtained by computing histograms from texture patterns. However, such processes ignore very important spatial information related to the texture features. This study designs a new feature vector, namely, K-spectrum, by using Baddeley’s K-inhom function to characterise the spatial distribution information of feature point sets. Texture features extracted by RIU4-LQP and K-spectrum are utilised to classify mammograms into BI-RADS density categories. Three feature selection methods are employed to optimise the feature set. In our experiment, two mammogram datasets, INbreast and MIAS, are used to test the proposed methods, and comparative analyses and statistical tests between different schemes are conducted. Experimental results show that our proposed method outperforms other approaches described in the literature, with the best classification accuracy of 92.76% (INbreast) and 86.96% (MIAS).

## 1. Introduction

Mammography screening is the most reliable and widely used method for observing breast lesions and evaluating breast health, and has been demonstrated to be effective for preventing breast cancer in its early detection stage. Breast density classification relates to measuring the amount of radiodense tissue (i.e., fibroglandular tissue) in mammograms and producing an important biomarker for indicating the risk of developing breast cancer in the future. Related clinical work shows that women with dense breast could face four- to six-fold higher risk than other women with low density [1]. Different metrics have been proposed and used to classify breast density, including six-class-categories (SCC) [2], Wolfe’s four categories [3], and breast imaging-reporting and data system (BI-RADS) [4]. BI-RADS criterion proposed by the American College of Radiology (ACR) has been widely used in clinical applications and includes four density categories: fatty (i.e., BI-RADS I), scattered density (i.e., BI-RADS II), heterogeneously dense (i.e., BI-RADS III), and extremely dense (i.e., BI-RADS IV). However, current clinical workflow heavily depends on radiologists’ subjective visual assessment, which is known to cause both inter- and intraobserver disagreements [5].

AI-driven classification algorithms have the potential to contribute to radiologists’ workflow by offering a secondary perspective of evaluating breast density. An automated breast density estimation method usually contains the following key steps: (1) extracting discriminative image features related to target density labels, (2) feature selection and parameter optimisation, (3) training a machine-learning-based classification model, and (4) predicting density labels for test images. As such, extracting effective features in mammograms plays an important role in obtaining accurate classification results. For breast density classification, relevant features can be broadly grouped into three types: heterogeneous features, deep-learning-based features, and texture descriptors.

Methods using heterogeneous features try to collect multiple image features, including image intensity, morphological features, entropy, texture features, etc., and combine them into one feature vector for classifying mammograms. Mario et al. [6] extracted mammogram image features based on image intensity, histograms, and grey-level co-occurrence matrix (GLCM). They also used Wrappers to select relevant features for improving the classification accuracy. Li et al. [7] extracted 137 pixel-level features containing intensity, GLCM, and morphological features, to group pixels into fatty or dense classes. In [8], intensity, texture, and morphological features were combined to develop a binary classification framework similar to that given in [7]. Statistical features including mean, standard deviation, smoothness, third moment, uniformity, and entropy were extracted in [9] to classify mammographic density into three groups. Qu et al. [10] proposed a fuzzy–rough refined image processing method to enhance local image regions and extracted GLCM-based statistical features for classifying breast density. Tzikopoulos et al. [11] extracted image features based on intensity and fractal texture, and SVM was used to classify mammograms into three categories. A multiresolution histogram method was used to analyse texture features in [12], and mammograms were classified into three density categories using a directed acyclic graph (DAG)–SVM classifier. In [13], different texture feature sets were investigated separately, including LBP, local grey-level appearance (LGA), textons (MR8 texton and image-patch texton), and basic image features (BIF). Their experimental results indicated that image-patch texton features performed better for four BI-RADS density categories classification. A lattice-based approach [14] was proposed to extract statistical and structural features for analysing parenchymal texture in mammograms.

Deep-learning methods have been used recently to analyse medical images for both classification and segmentation tasks with promising results. For evaluating breast density, convolutional neural networks (CNN) were applied to manage binary classification tasks. An AlexNet-based CNN model was proposed in [15] to distinguish two BI-RADS categories (‘scattered density’ and ‘heterogeneously dense’). Ahn et al. [16] designed a CNN architecture to extract features from mammogram patches and to classify them into dense and fatty groups. In [17], a fully convolutional network (FCN) was used to segment breast region and fibroglandular areas, based on which a percentage density was estimated. A deep convolutional neural network (DCNN) was used to classify mammographic pixels into fatty or dense class in [7]. Li et al. [18] proposed a CNN-based radiomics method combined with dilated convolutions and attention mechanisms to extract high-throughput features that were used to classify mammograms into four BI-RADS density categories. One limitation of applying deep-learning methods for mammogram classification task is that they require a huge number of training images with accurate annotations by clinicians [19].

Recent study using texture descriptors such as local binary patterns (LBP) and their variants have shown promising classification performance with over 80% accuracy. The local binary patterns (LBP) method was first proposed in [20] to describe image texture patterns. Owing to its simplicity and efficiency, LBP has been studied widely and new variants were proposed for extracting texture features and classifying medical images. In [21], LBP was extended to elliptical LBP (ELBP) and mean-elliptical LBP (M-ELBP) by considering various neighbourhood topologies and different local region scales. M-ELBP presented a desirable classification result (77.38 ± 1.06) on an MIAS dataset. Tan et al. [22] proposed local ternary patterns (LTP) using a three-value encoding approach compared to two-value encoding of LBP. Then, Rampun et al. [23] extracted LTP-based texture features to classify MIAS mammograms into four BI-RADS categories. Nanni et al. [24] proposed local quinary patterns (LQP) by extending LBP from a binary encoding system to a five-value encoding system, and used three different medical image classification tasks to test this new texture descriptor. Subsequently LQP was investigated and extended with multiresolution and multiorientation schemes in [25] to classify mammographic density. Their experimental results have shown that the use of LQP-based texture features helps improve the overall classification accuracy. In a recent study, Rampun et al. [26] tried the local septenary patterns (LSP) method by using seven-value encoding approach to further improve the classification performance. Their experimental results demonstrated that classification accuracy was slightly improved by using LSP compared to LQP (80.5 ± 9.2 vs. 80.1 ± 10.5 on INBreast), but this improvement was not statistically significant (*p* = 0.45).

Through reviewing relevant feature extraction methods above, we can find that multilabel (three or four) classification is challenging work with only a few reported results surpassing 80% accuracy. The work in [11,12,21] reported accuracy around 77% for classifying MIAS mammograms into three density categories; experimental results in [6,13] showed 79% and 75% accuracy for four categories classification on the same dataset. A recent study based on texture analysis using LBP variants has shown promising classification performance with over 80% accuracy. LTP, LQP and LST were used in [23,25,26] and obtained the accuracy of 82%, 86%, and 83%, respectively, with four density categories classification on MIAS. When testing the INbreast dataset, classification accuracy of 82.02% and 80.10% were seen by using LQP-based texture features [25,26].

However, the use of LBP and its variants for feature extraction have some limitations that can affect the accuracy of results: (1) For capturing more local texture information, increasing the number of neighbouring pixels and a multiscale scheme are usually used, but they lead to an exponential increase in the number of features (i.e., high feature dimensionality). (2) The conventional rotation invariant uniform method (i.e., ‘RIU2′) can reduce the high feature dimensionality, but it also suppresses some important/distinguishing features and lowers the accuracy of classification results. (3) Most approaches in the literature extract texture information by computing histograms. Histograms offer quantity information of features but ignore their spatial distribution characteristics, which may contain other important and complementary information related to mammographic density. (4) Related work in this field did not give much attention to feature optimisation after collecting the initial feature set, while a proper feature selection method could optimise the feature vector and improve the accuracy of results.

Motivated by relevant work and a comparison of their performance characteristics, this study chooses LQP as the base feature extraction method, and two improvement schemes called RIU4-LQP and K-spectrum are proposed to develop a more robust and efficient texture descriptor for mammogram analysis. Focusing on the breast density classification task, the main contributions of this paper include:

1. A novel texture descriptor, namely, rotation invariant uniform local quinary patterns (RIU4-LQP), is proposed by developing a uniform rotation invariant version of LQP and considering a higher number of bit transitions while computing the invariant descriptors. To the best of our knowledge, this is the first study that extends the uniform encoding technique by using the transition number of four.
2. In addition to LQP feature histograms, we address a spatial feature extraction framework using Baddeley’s K-inhom function, which outputs a new feature vector called K-spectrum.
3. A new feature space is proposed by concatenating RIU4-LQP histograms and K-spectra and is used in the mammographic density classification model.
4. Machine-learning-based feature selection methods are employed to optimise the initial texture feature set, which does not only reduce the high feature dimensionality but also lead to a better classification performance.
5. We empirically proved the effectiveness of the proposed classification model on two publicly available mammogram datasets. Experimental results indicate that the extra spatial distribution features considered in this work are beneficial to the improvement of the classification accuracy.

The remaining part of this paper is organised as follows. Section 2 introduces the datasets used in this study. Research methods including the development of pre-processing methods, feature extraction schemes, feature selection algorithms, and the classification model are described in Section 3. Parameter optimisation is introduced in Section 4. Experimental results and discussion are given separately in Section 5 and Section 6. Section 7 summarises the paper.

## 2. Materials

To test the proposed methods, two mammogram datasets INbreast [27] and MIAS [28] containing different image types and using different density classification criteria, are used in our experiments.

INbreast [27] is a full field digital mammogram (FFDM) dataset and consists of 410 images from 115 cases, including bilateral mediolateral oblique (MLO) and craniocaudal (CC) views. Each image is saved in the DICOM format and in size of 3328 × 4084 or 2560 × 3328 pixels, depending on the compression plate used in the acquisition. This dataset offers carefully associated ground truth (GT) annotations made by a specialist in the field and validated by a second specialist. For each mammogram, its density is labelled as one out of four BI-RADS categories (BI-RADS I-IV). Images distribution with their density labels are as follows: 136 (BI-RADS I), 146 (BIRADS II), 99 (BI-RADS III), and 28 (BI-RADS IV).

**Figure 1:**
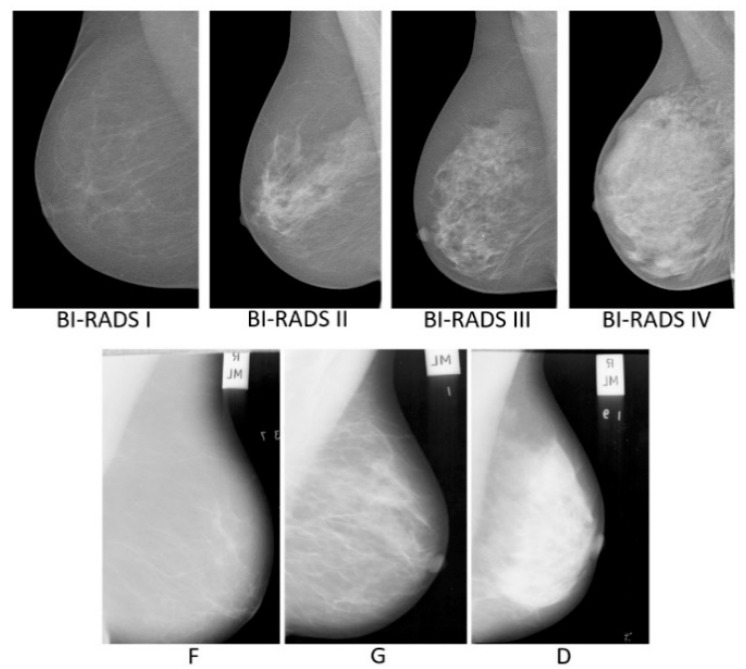
First row: INbreast mammograms in four BI-RADS density classes. Second row: MIAS mammograms in three tissue density classes.

MIAS [28] is a scan field mammograms (SFM) dataset, containing 322 images taken from 161 women, with only MLO views on both sides from the UK National Breast Screening Programme. Each mammogram is at 50 micron resolution in portable grey map (PGM) format. All images are associated with density ground-truth labels of three classes: fatty (F), fatty-glandular (G), and dense-glandular (D). There are 106 images belonging to fatty group, 104 and 112 images to the fatty-glandular and dense-glandular classes. Figure 1 shows sample images belonging to each density category from the two datasets.

## 3. Methodology

**Figure 2:**
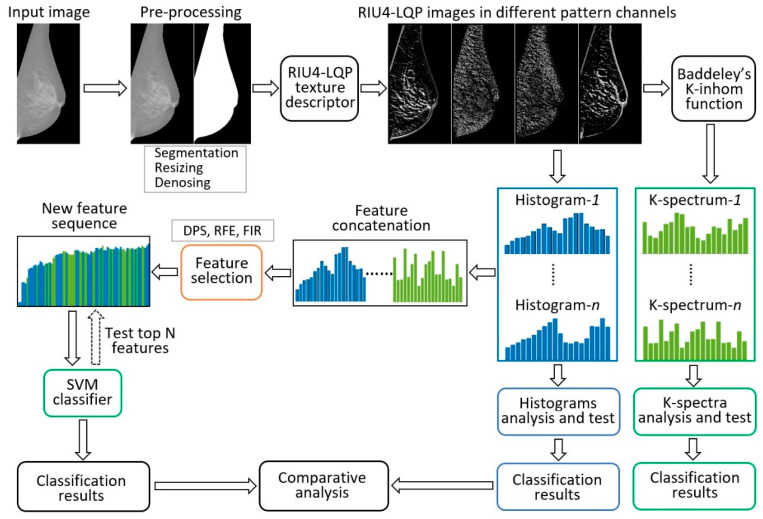
An overview of the breast density classification process using the proposed methods.

This paper proposes a novel texture descriptor called rotation invariant uniform local quinary patterns (RIU4-LQP) to describe texture patterns in mammograms. In addition to histogram information, this study further explores richer statistical information extracted from the RIU4-LQP feature set by using Baddeley’s K-inhom method [29]. A new feature vector called K-spectrum is developed based on the extracted spatial information, which offers supplementary and important image features. Histograms and K-spectra are first extracted and tested separately and then are combined together to create a new texture feature space. A novel feature selection step is considered carefully in this study for removing redundant image features. An overview of the workflow using our proposed methods is shown in Figure 2, and more details are given in the following sections.

### 3.1. Pre-Processing

**Figure 3:**
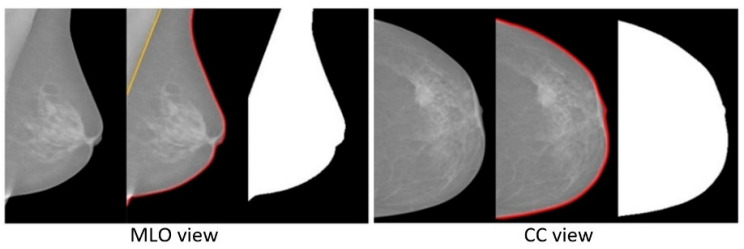
Examples of segmenting breast region and removing pectoral muscle area in INbreast mammograms.

**Figure 4:**
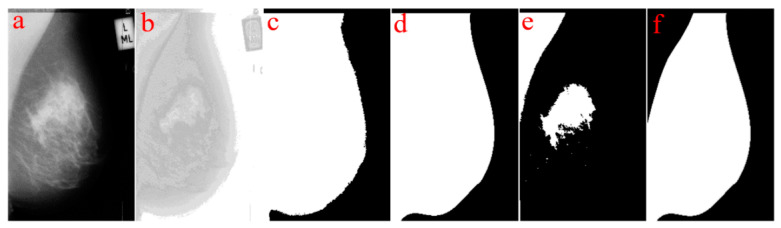
Segmentation of breast region in MIAS mammograms: (**a**) input mammogram, (**b**) enhancing breast region and artefact areas, (**c**) rough contour of breast region, (**d**) smoothing breast contour, (**e**) finding pectoral muscle area, (**f**) breast mask image without pectoral muscle region.

**Figure 5:**
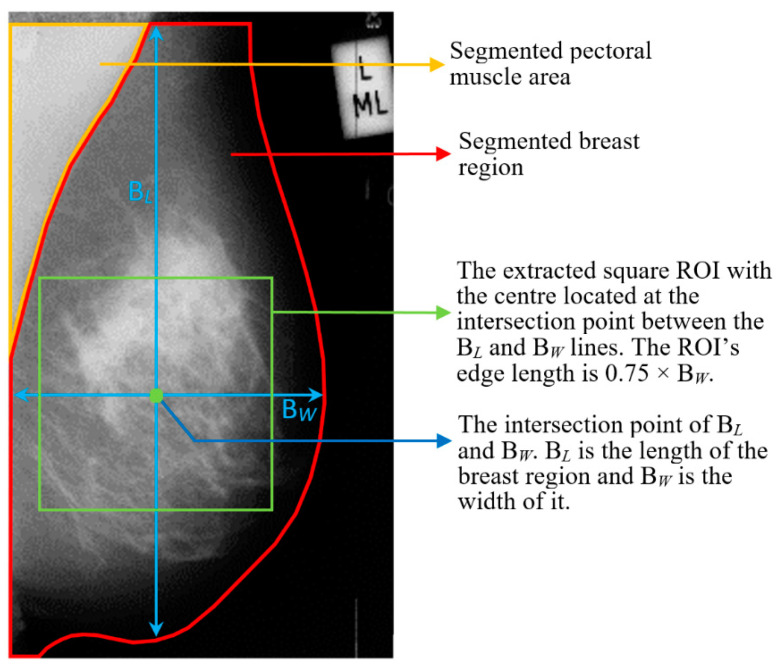
ROI extraction from the central area of MLO-view mammogram in MIAS dataset.

This pre-processing step contains breast area segmentation, denoising operation, and resizing the image. Breast segmentation is first applied to remove nonbreast areas such as background region, pectoral muscle, and label artefacts. A multifractal-method-based feature-enhanced image [30] is used to highlight the contrast between image background and the breast tissue region. The intensity thresholding method and morphological operations [31] are used to separate breast region and artefacts from the background. The label artefacts can be removed by keeping only the largest connected area (breast region). K-means algorithm and polynomial fitting approach [32] are employed to eliminate pectoral muscle from the breast region in MLO view mammograms. A median filter of 3 × 3 size is used to reduce noise. Finally, mask images are obtained, which are used to extract image features only from the region of interest (breast area) in the following steps. As relative work [16,17] reported promising classification results using resized mammogram images, this study applies the bicubic interpolation method to resize processed images with a scale factor s [33], resulting in a resized image that is s times the size of original image. Figure 3 and Figure 4 show some examples of segmenting breast region from image background using INbreast and MIAS mammograms, respectively. For the MIAS dataset that only contains MLO view images, relative work [21,34] demonstrated that using a cropped square region of interest (ROI) produces a better classification result. This study therefore uses a similar method as that in [34] to obtain the ROIs in MIAS. Figure 5 illustrates ROI extraction in MIAS mammograms. A detailed introduction of this pre-processing stage can be found in Supplementary Materials.

### 3.2. Local Quinary Patterns (LQP) and RIU4-LQP

**Figure 6:**
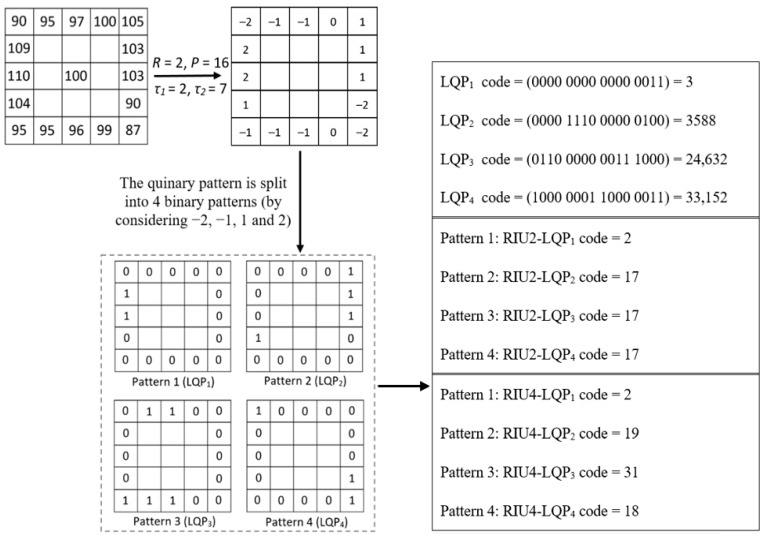
LQP encoding example, one 5-value pattern is split into 4 LBP patterns.

Ojala et al. [20] proposed LBP to describe local image structure and to extract texture features. Nanni et al. [24] proposed LQP by extending LBP from a binary value encoding scheme to a 5-value encoding algorithm. LQP uses values −2, −1, 0, 1, and 2 to describe the relations between the intensity values of a central point and its neighbours. Each LQP code can be split to 4 LBP patterns, capturing more detailed texture information. Thus, for analysing medical images such as mammograms that contain regions of subtle texture differences, texture features extracted by LQP are more informative to be used to improve the classification accuracy. To implement the LQP operator, two threshold values {*τ*_1_*, τ*_2_} are required for generating a 5-value encoding pattern. The calculation of LQP code can be described as follows:

(1) $$LQP_{i}(c,\;R,\;P)\;=\;\sum_{p=0}^{P-1}s_{i}(I_{p}\;-\;I_{c})2^{p},\;i=1,\;2,\;3,\;4$$

(2) $$s_{1}(x)=\{\begin{array}{c} 1,\;if\;d(x)=2\\ 0,\;otherwise\; \end{array}$$

(3) $$s_{2}(x)=\{\begin{array}{c} 1,\;if\;d(x)=1\\ 0,\;otherwise\; \end{array}$$

(4) $$s_{3}(x)=\{\begin{array}{c} 1,\;if\;d(x)=-1\\ 0,\;otherwise\; \end{array}$$

(5) $$s_{4}(x)=\{\begin{array}{c} 1,\;if\;d(x)=-2\\ 0,\;otherwise\; \end{array}$$

(6) $$d(x)=\{\begin{array}{cc} 2, & \tau_{2}\leq x\;\\ 1, & \tau_{1}\leq x<\tau_{2}\;\\ 0, & -\tau_{1}\leq x<\tau_{1}\;\\ -1, & -\tau_{2}\leq x<-\tau_{1}\\ -2, & x<-\tau_{2}\; \end{array}$$

where *R* denotes the radius of a circular neighbourhood of the centre pixel *c* and *P* is the number of neighbourhood pixels used to calculate LQP code. *I_p_* and *I_c_* are intensity values of the *p*th neighbour pixel and the centre pixel c, respectively. From a specific position (usually the top-left corner) the binary values given by *s_i_(x)* are gathered in a specific sequence (usually in clockwise order) to obtain the LQP_i_ codes. Figure 6 illustrates how the LQP operator works in a local region with *R* = 2 and *P* = 16. After obtaining the 5-value pattern, it is split into 4 binary patterns by *s_i_(x)* in Equations (2)–(5).

LQP produces a high feature dimensionality given by 2*^P^*^+2^, which increases exponentially with *P*. A large feature space cannot be utilised efficiently to train a classification model, and information redundancy in the feature set can have a negative impact in the final classification performance. To resolve the high dimensionality problem, one option is to use a rotation invariant strategy to extend LQP to rotation invariant uniform LQP (RIU2-LQP) [35]. However, RIU2-LQP suppresses too much texture information (from 2*^P^* ^+ 2^ to 4 × (*P* + 2)) which may in turn reduce the final classification performance (Figure 6).

Under the consideration discussed above, this paper extends LQP to RIU4-LQP by analysing the transition number in a wider range. RIU4-LQP allocates different codes to binary patterns with two ‘1′-contiguous segments (e.g., patterns 2–4 in Figure 6), which captures richer image representations of texture patterns compared to RIU2-LQP. The proposed RIU4-LQP encoding scheme can be formulated by following equations:

(7) $$LQP_{i}^{riu4}(c,R,P)=\{\begin{array}{cc} \sum_{p=0}^{P-1}s_{i}(I_{p}-I_{c}), & if\;T_{i}(LQP(c,R,P))\in \left\{0,\;2\right\}\\ \;P+index, & if\;T_{i}(LQP(c,R,P))=4\;\\ \left\lceil \frac{P^{2}+11}{4}\right\rceil -1, & otherwise\; \end{array}$$

(8) $$T_{i}(LQP(c,R,P))=\left|s_{i}(I_{P-1}-I_{c})-s_{i}(I_{0}-I_{c})\right|\;+\;\sum_{p=1}^{P-1}\left|s_{i}(I_{p}-I_{c})-s_{i}(I_{p-1}-I_{c})\right|$$

(9) $$index=\{\begin{array}{cc} \sum_{n=1}^{X-1}(P-3-2(n-1))+(Y-X+1), & if\;X\geq 2\\ \;Y-X+1, & if\;X=1 \end{array}$$

where *i* ∈ {1, 2, 3, 4}; *T_i_*(*·*) is defined as the number of spatial transitions (bitwise 0/1 changes) in patterns. *X* and *Y* denote the number of occurrences of ‘1′ (defined by *s_i_(x)*) in two distinct contiguous segments when *T* = 4 (e.g., Figure 6, pattern 2–4). We impose a restriction with respect to the relationship between *X* and *Y*: *X* always denotes the shorter ‘1′-contiguous segment, i.e., *X* ≤ *Y*, for example, the pattern-3 in Figure 6: *X* = 2, *Y* = 3. Equation (7) contains three parts to correspond to different bit transitions (i.e., the value of *T*) for recognising and encoding texture patterns. The first part (i.e., the first row of Equation (7)) is same with RIU2-LQP for distinguishing texture patterns that have none or only one ‘1′-contiguous segment (e.g., the pattern-1 in Figure 6). The second part of Equation (7) aims to encode texture patterns with two ‘1′-contiguous segments (e.g., patterns 2–4 in Figure 6). As the encoding values from 0 to *P* have been allocated with the condition of *T* ∈ {0, 2}, the output code starts from *P* and adds the value of *index*, which counts from 1 using Equation (9). The value of *index* is used to recognise and label those texture patterns when *T* = 4, for example, *index* = 1 with the pattern of *X* = 1, *Y* = 1; *index* = 2 with the pattern of *X* = 1, *Y* = 2; and so on. The third part of Equation (7) uses a unified code to denote all the other texture patterns. By using the proposed RIU4-LQP encoding method, RIU4-LQP codes are allocated to different texture patterns shown in Figure 6.

### 3.3. Rotation Invariant Property of RIU4-LQP and Its Utilisation

**Figure 7:**
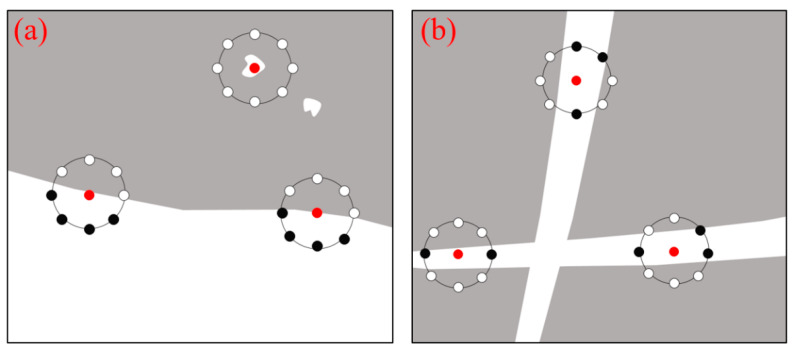
Different local texture structures are described by LQP variants using 8 neighbour pixels *I_p_* with the central pixel *I_c_* marked in red. Black points represent ‘0′ in the circular bit patterns and white points represent ‘1′. (**a**) Microstructures of spots and edges can be captured by both RIU2-LQP and RIU4-LQP. (**b**) Microstructures of thin stripes are effectively represented by RIU4-LQP but not by RIU2-LQP.

The basic LQP method is not rotation invariant. When the processed image is rotated, the neighbour pixels *I_p_* will correspondingly move along the perimeter of the circle around *I_c_* (Figure 7). Since the binary coding sequence of LQP always begins from a pre-designated and fixed position, e.g., to the top-left of *I_c_*, rotating a particular binary pattern naturally results in a different LQP code.

To remove the effect of rotation, the function *T_i_*(·) that measures bit transitions is used in Equation (8) for defining uniform patterns. In our proposed variant, the uniform patterns refer to circular structures that contain extended spatial transition conditions (i.e., *T* = 0, 2, 4, or others), such as patterns shown in Figure 6 with *T* = 2 or 4. By using *T_i_*(*·*) and Equation (7), the encoding system works by recognising different ‘1′-sequence patterns without considering a fixed binary order, thus achieving rotation invariance. For example, when *T* = 2, the binary sequences ‘00111100′ and ‘00001111′ share the same RIU4-LQP code of 4, presenting a microstructure of an edge, while in the basic LQP, they have two different code values of 60 and 15. Similarly, when *T* = 4, the sequences of ‘11001110′ and ‘11101100′ share the same RIU4-LQP code of 15, as the related ‘1′- sequence patterns are same (with *X* = 2, *Y* = 3 using Equation (9)). In addition, under the condition of *T* = 4, some microstructures presenting thin stripe shapes with two-sided edges are captured by RIU4-LQP, but failed to be represented by LQP or RIU2-LQP. In mammograms, such local structures are commonly observed in regions with fibroglandular tissues that relate to breast density. Figure 7 illustrates the typical texture structures captured by RIU4-LQP. Based on this design, we assume that the proposed RIU4-LQP possibly captures more structural details for mammographic images, and this argument is tested and demonstrated in the following experiments.

**Figure 8:**
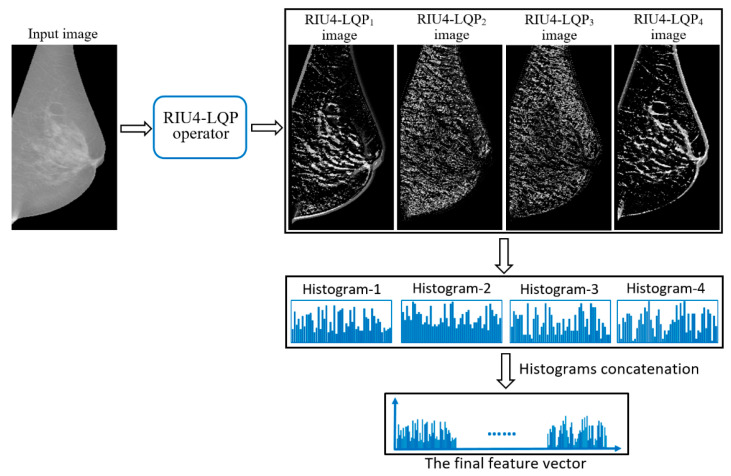
Example of RIU4-LQP images in different texture pattern channels and corresponding feature vectors.

The proposed RIU4-LQP reduces the feature dimensionality from 2*^P^* ^+ 2^ to *P*^2^ + 11. Meanwhile, compared to RIU2-LQP, more texture patterns are included in the extracted features by considering a higher number of bit transitions. Figure 6 gives the comparison between RIU2-LQP and RIU4-LQP codes on same patterns. Figure 8 shows RIU4-LQP images in different texture pattern channels by substituting corresponding RIU4-LQP codes for pixel intensities and their histogram-based feature vectors.

### 3.4. Baddeley’s K-Inhom Function

**Figure 9:**
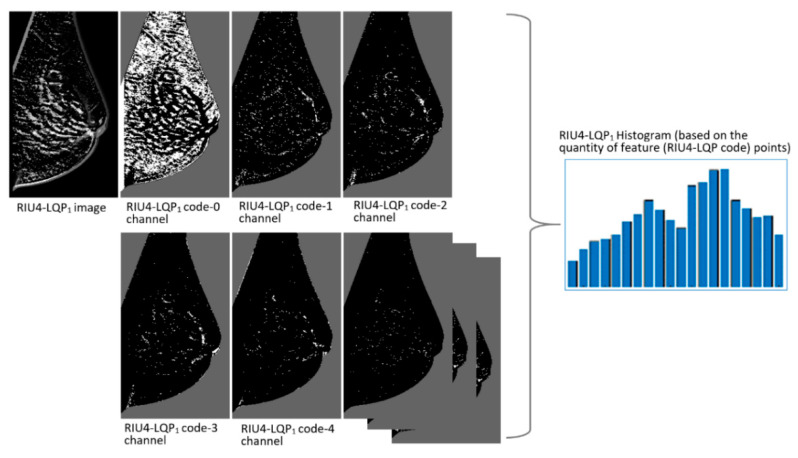
Decomposition of the RIU4-LQP_1_ image in Figure 8 based on each encoding channel. The positions of pixels in each RIU4-LQP_1_ code channel considered are marked by white points in the black breast region background.

**Figure 10:**
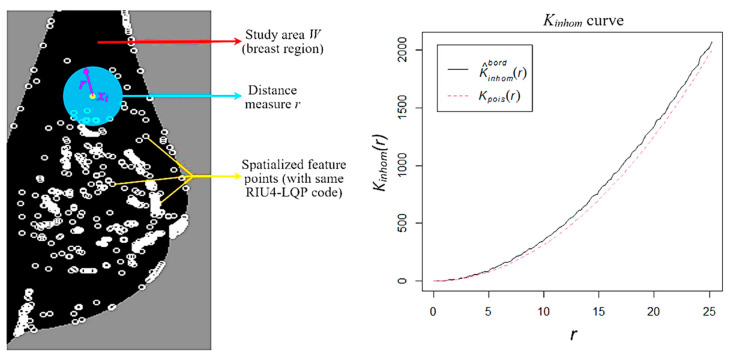
One feature pattern image (**left**) in a RIU4-LQP code channel with all feature points emphasised by white circles; the corresponding Kinhom curve (**right**) is generated by Baddeley’s K-inhom function.

In conventional applications of texture descriptors [21,25,26,34], image texture features are represented by histogram information that counts the quantity of feature points but ignores their spatial distribution characteristics (Figure 9). This study employs Baddeley’s K-inhom method [29] to extract spatial information based on RIU4-LQP feature points. The K-inhom method is a variant of Ripley’s K function [36], and both are statistical analysis schemes used for studying qualitative or quantitative characteristics of spatialised data. Generally, K-inhom function for one-dimensional data can be described as follows:

(10) $$K_{\mathrm{inhom}}(r)=\frac{1}{D}\sum_{i}\sum_{j\neq i}\frac{1\{\left\|x_{i}-x_{j}\right\|\leq r\}}{\hat{\lambda }(x_{i})\hat{\lambda }(x_{j})}c(x_{i},x_{j};r)$$

(11) $$D=\frac{1}{\left|W\right|}\sum_{i}\frac{1}{\hat{\lambda }(x_{i})}$$

(12) $$c(x_{i},x_{j};r)=\frac{1\{b_{i}>r\}}{\sum_{j}^{}\left(1\{b_{j}>r\}/\hat{\lambda }(x_{j})\right)}$$

where 1{||*x_i_*−*x_j_*|| ≤ *r*} denotes an indicator that takes a value 1 if the distance between point *x_i_* and point *x_j_* is less than or equal to *r* or 0; otherwise, *r* is a distance measure; *c*(*x_i_*; *x_j_*; *r*) corresponds to the correction of edge effects and *W* to the region of interest; and *b_i_* is the distance from *x_i_* to the boundary of *W*. The function *c*(*x_i_*; *x_j_*; *r*) is implemented as in [37] for border corrections. *λ*(*x_i_*) denotes an intensity function around point *x_i_*, which is defined by the number of neighbour points (*x_j_*) expected in a small area (using the radius of *r*) with *x_i_* in the centre, but *λ*(*x_i_*) is not known in practice. Instead, ‘$\hat{\lambda }\left(x_{i}\right)$’ is used in Equations (10) and (11) as the estimation of *λ*(*x_i_*), which is implemented by a nonparametric method [29]. More details concerning Baddeley’s K-inhom function and its implementation can be found in [29,38]. The ‘spatstat’ package [38] in R is used to implement Baddeley’s K-inhom method. Values of *K_inhom_*(*r*) are calculated with different distance measurements (*r*), which result in a *K_inhom_* curve by connecting all the observed values of *K_inhom_*(*r*) (Figure 10). In [37,38], an expected reference value *K_pois_*(*r*) = *πr^2^* (the red dotted line in Figure 10) obtained based on an inhomogeneous Poisson process, is used to compare with the observed *K_inhom_*(*r*) value. If the *K_inhom_* curve is located below the reference curve (i.e., *K_inhom_*(*r*) < *K_pois_*(*r*)), it indicates that corresponding points scatter regularly in the region of interest. By contrast, if the *K_inhom_* curve is located above the reference curve (i.e., *K_inhom_*(*r*) > *K_pois_*(*r*)), the distribution of points tends to be more aggregated. Therefore, the *K_inhom_* curve can be used to describe the spatial distribution characteristics of a point set. As this paper focuses on mammogram image analysis, we use the segmented breast region as the region of interest *W* (Figure 10). The pixels decomposed by RIU4-LQP code values (Figure 9) constitute feature point sets in different code channels, which produce their *K_inhom_* curves separately and show corresponding spatial characteristics.

### 3.5. K-Spectrum of RIU4-LQP

This paper proposes a new texture feature vector called K-spectrum that is based on the *K_inhom_* curve representing the spatial distributions of texture feature point sets in mammograms. The RIU4-LQP operator is used to produce a RIU4-LQP*_i_* code set {*code-1, code-2, …, code-k*}. Subsequently, pixels in breast region *W* are decomposed into *k* different point sets {*X*_1_*, X*_2_*, …, X_k_*} by corresponding RIU4-LQP*_i_* code values (Figure 9). Baddeley’s K-inhom function is adopted to output a *K_inhom_* curve for each point set *X_i_*, which reflects how these points are scattered in the breast region with respect to a specific distance measure (*r*). As introduced in the last section, a reference curve *K_pois_(r)* is used for comparing with the observed *K_inhom_* curve. Therefore, this study uses a deviation (*d*) between the observed *K_inhom_(r)* value and the reference value *K_pois_(r)* under the radius *r* and its mean ($\bar{d}$) on a valid *r* range to evaluate the spatial distribution information of point sets. The deviation *d* and the mean $\bar{d}$ are computed as follows:

(13) $$d\left(r\right)=K_{inhom}\left(r\right)-K_{pois}\left(r\right)$$

(14) $$K_{pois}\left(r\right)=\pi r^{2}$$

(15) $$\bar{d_{i}}=\frac{\sum_{r=1}^{s}d_{i}(r)}{s},\;i=1,2,\ldots ,k.$$

**Figure 11:**
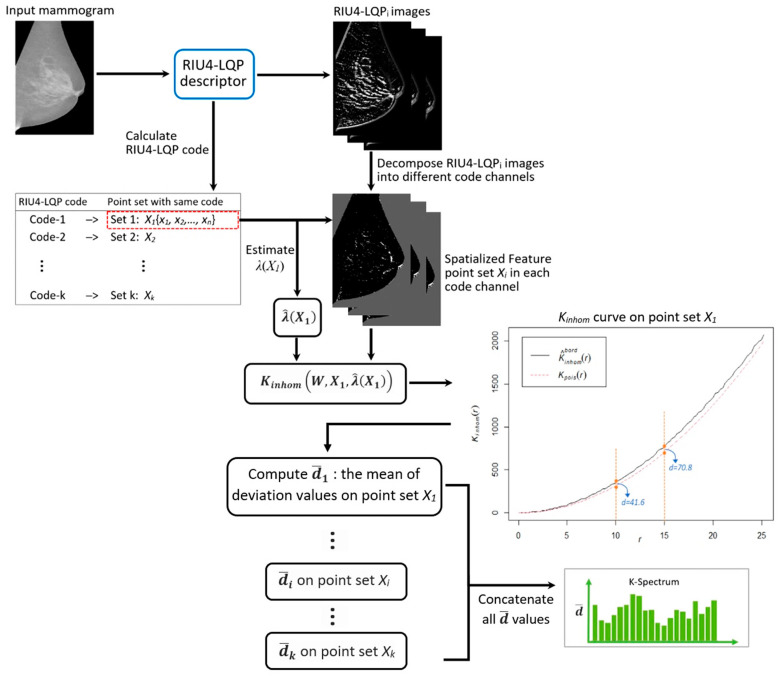
Illustration of using the K-inhom method and RIU4-LQP operator to generate the new feature vector (K-spectrum).

All the means (${\bar{d}}_{1},\;{\bar{d}}_{2},\ldots ,\;{\bar{d}}_{k}$) are concatenated to form a new feature vector called ‘K-spectrum’. Figure 11 shows how the K-inhom function works in mammogram images with the proposed procedures and generates the K-spectrum.

### 3.6. Feature Selection

As introduced in the first section, the initial feature space usually is too large to be used efficiently, and it is quite likely that some redundant features are contained within it. Therefore, a feature selection step is necessary to optimise the initial feature set. This study investigates three feature selection methods: dominant patterns set (DPS) [39], recursive feature elimination (RFE) [40], and feature importance ranking (FIR) [41].

Guo et al. [39] proposed a dominant patterns set (DPS) method to construct a subset of the initial feature set for filtering the most frequently occurring feature patterns. This feature selection method has been used in [25,26] for reducing feature dimensionality. In DPS, a set of dominant patterns of an image is defined as a minimum set that can cover *n*% (0 < *n* < 100) of all patterns. To produce a subset, a bin-wise summation for all the histograms of image features in a training set is performed first and this results in a histogram (*H*) with *U* bins. Then, all the bins of histogram (*H*) are sorted in descending order, and top *M* bins are selected using Equation (16):

(16) $$\arg\underset{M}{\min}\frac{\sum_{i=1}^{M}H(i)}{\sum_{j=1}^{U}H(j)}\geq n\%$$

where *U* is the bins number in *H* and *n* is a threshold value by which *M* dominant patterns are selected from *H*.

The RFE [40] method recursively removes features and builds a model on remaining features. The model accuracy is used to identify which features contribute more than others for predicting the target classes. The estimated best feature is assigned a rank score ‘1′, and the least related features have the highest rank sores.

The FIR method [41] uses ensembles of decision trees (e.g., random forest) to compute the relative importance of each attribute. An importance score is given for each feature to indicate that certain features play more important roles than others in the class prediction.

All feature selection methods produce a new sequence of features ranked according to their relevance/importance. The top (best) *N* features in this sequence can be selected and used to test the corresponding classification performance with the training set, and an *N*-feature set with the highest accuracy is the final feature vector used for the test set.

### 3.7. Classification

As related work [9,11,23,25,26] reported their best classification results by using SVM to predict target labels of mammographic density, SVM is used in this study for training the classification model and producing classification results on test images. Since this classification work aims to classify mammograms into multiple density categories (three or four), a multiclass SVM that is implemented by the one against all (OAA) method is used in this model. To obtain the optimum classification results, the three kernels used most often, RBF, Poly, and Sigmoid, are tested in this work. The grid-searching method is used to find the best combinations of parameters (*gamma*, *C*, and *degree*).

## 4. Parameter Optimisation

The methods used in the proposed classification model involve several parameters that affect the classification results differently. This section summarises the relevant parameters in different processing steps and addresses the test and optimisation methods.

### 4.1. Relative Parameters

The parameters and the range of their initial values are listed in Table 1. In the pre-processing stage, a scaling parameter *s* is selected from a set of {1, 1/2, 1/4, 1/8, and 1/16} by testing different mammogram datasets. Comparative analysis is conducted in our previous work [33] and the same settings of *s* are used. When using RIU4-LQP to extract the feature set, a multiscale strategy is used for capturing richer image representation. Referring to related work in [25], three pairs of (*R, P*) with corresponding settings shown in Table 1 are used. The LQP-based method needs two extra threshold values {*τ*_1_*, τ*_2_} in its encoding system, which requires manual determination. An automatic approach is proposed in [42] by considering central pixel’s intensity (*I_c_*). Similarly, we introduce the following empirical rules for adaptively deciding *τ*_1_ and *τ*_2_: *τ*_1_ = *I_c_* × 2% and *τ*_2_ = *I_c_* × 7%. For calculating the K-spectrum feature vector, different values of the distance measure (*r*) are used to produce the *K_inhom_* curve (i.e., the *K_inhom_* curve in Figure 10). Valid *r* values rely on the region of interest (*W*) (i.e., the segmented breast region), which does not have a uniform size among different mammograms. After comparing the observed *r*-ranges on all images in the dataset, a maximum valid *r*-range 1~25 is found and used to generate K-spectra. In the feature selection step, as the concrete values of (*R_i_, P_i_*) of RIU4-LQP are designated in Table 1, the feature dimensionality (*n*_1_ and *n*_2_) of feature sets can be obtained by Equations (7)–(9).

### 4.2. Selection of r-Range in K-Spectrum

**Figure 12:**
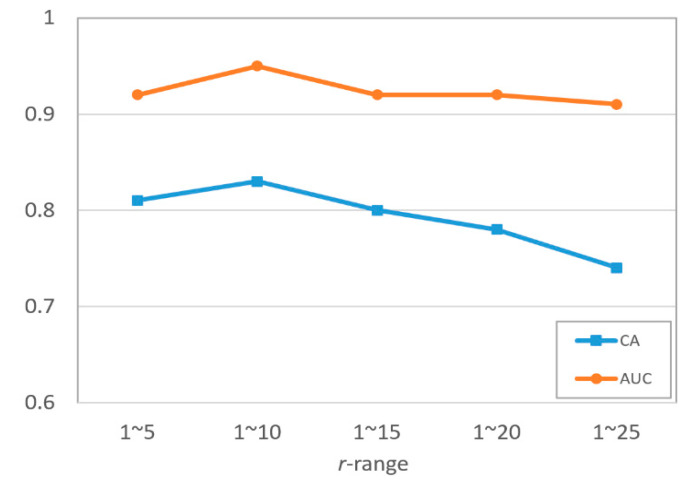
Comparison of classification performance on INbreast training set by using different value ranges of distance measurement *r*.

As shown in Table 1, the maximum valid *r* value range 1~25 is extracted when applying Baddeley’s K-inhom method in mammograms. Since there is no guarantee of the maximum range that is most effective for K-spectrum features, we narrow this maximum *r*-range by five-unit intervals, and five subranges are generated and tested for obtaining the optimal K-spectrum. Figure 12 shows that the highest classification accuracy (CA) is 0.83 and the highest AUCROC is 0.95 by using the *r*-range of 1~10 on INbreast dataset. Therefore, we use this *r* value range as the optimum distance measurement for producing K-spectrum in the following experiments.

### 4.3. Grid-Searching Results for SVM Classifier

**Figure 13:**
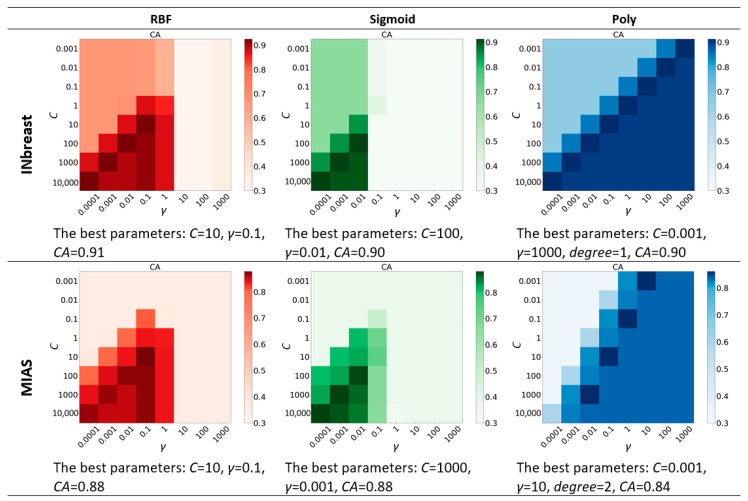
Heatmaps of grid-searching results using training sets on two datasets.

We consider three kernels (RBF, Sigmoid, and Poly) and different value ranges of other parameters (*gamma*, *C*, and *degree*) for the SVM classifier, as given in Table 1. The best combination of the kernel and parameters are found by grid-searching on two datasets (Figure 13).

## 5. Experiment and Results Analysis

As mentioned earlier, two mammogram datasets, INbreast and MIAS, are used to test the proposed classification model. To give a comprehensive and objective evaluation towards the classification performance, different measure criteria are used. For each test method, classification accuracy (CA) and area under the ROC curve (AUCROC) are calculated as the main performance indices. Since this study investigates the effectiveness of different feature selection methods, the final selected features number (*N*) is considered. We also conduct two different test methods: leave-one-woman-out test [11,13] and 10-fold cross validation [21,23,25].

### 5.1. Classification Results Using Histogram Information

**Figure 14:**
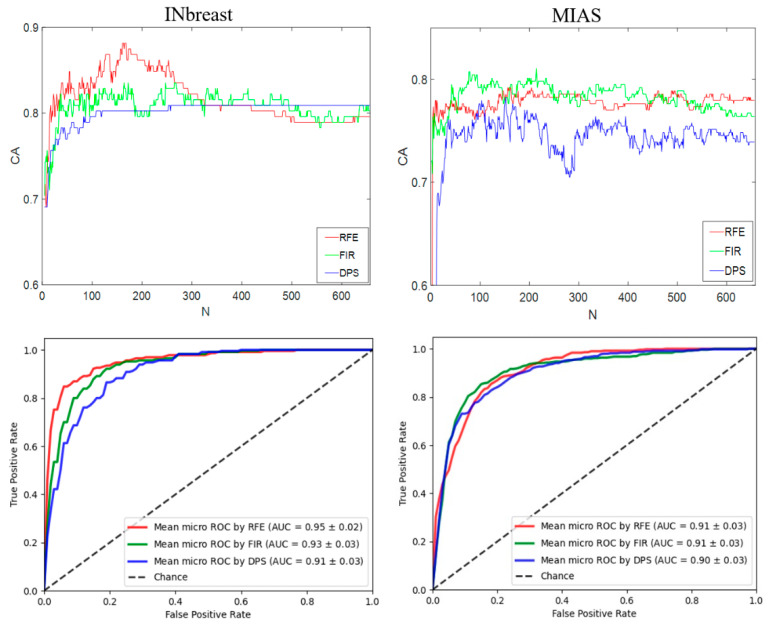
Classification accuracy (**first row**) and AUCROC values (**second row**) on two datasets using the feature vector based on histograms.

A histogram-based feature vector that contains 656 RIU4-LQP features is tested first on two datasets. Three feature selection methods (Section 3.6) re-sort the feature vector based on their importance. For each test iteration, a feature subset containing the first N features {*f*_1_*, f*_2_*, …, f_N_*} (*N* ≤ 656) is sent to the classifier for producing the classification result. Then, *N* is increased and the test procedure enters the next iteration, finally obtaining the curves of CA vs. N. Figure 14 shows the classification results on two datasets in which the highest CA is 88.16% and 81.06% on INbreast and MIAS, respectively. Meanwhile, we can also notice that different feature selection methods affect the classification results differently. For INbreast, RFE reduces the feature number *N* to 161 and obtains the highest AUCROC value (0.95 ± 0.02); for MIAS, the highest AUCROC value is 0.91 ± 0.03 when using FIR with *N* = 214.

### 5.2. Classification Results Using K-Spectrum

**Figure 15:**
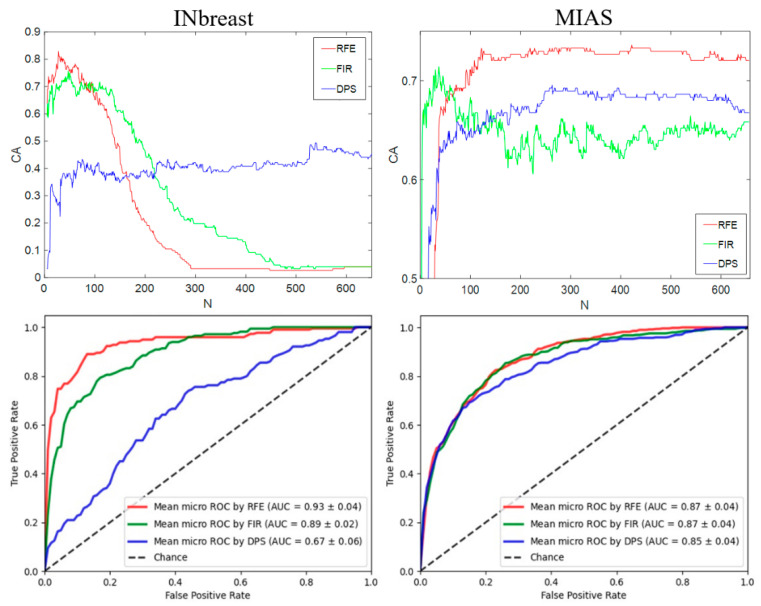
Classification accuracy (**first row**) and AUCROC values (**second row**) on two datasets using the feature vector based on K-spectra.

K-spectra are extracted in mammograms in two datasets and used as texture features in the classification model. As the extraction of K-spectra are based on the same RIU4-LQP operator and multiscale method used for collecting histogram information, the K-spectrum-based feature vector also contains 656 features. Classification performance is shown in Figure 15, with the highest CA of 82.89% and 73.60% obtained on two datasets.

### 5.3. Classification Results Using Concatenated Features

**Figure 16:**
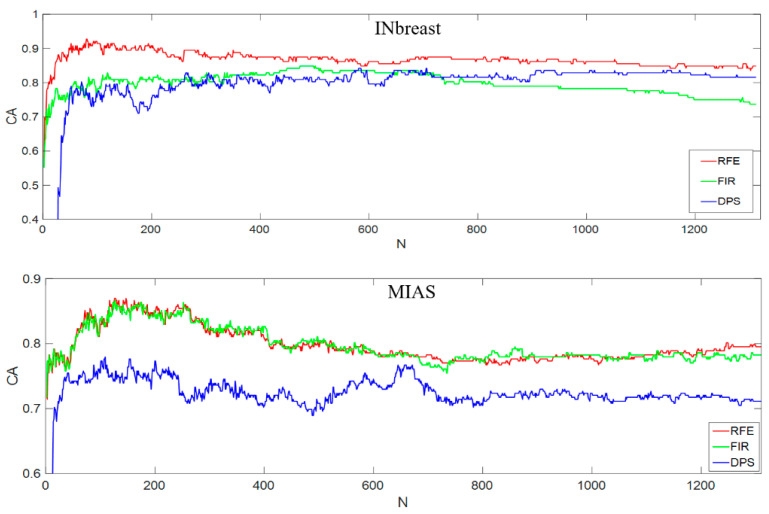
Classification accuracy on two datasets using combined texture features.

**Figure 17:**
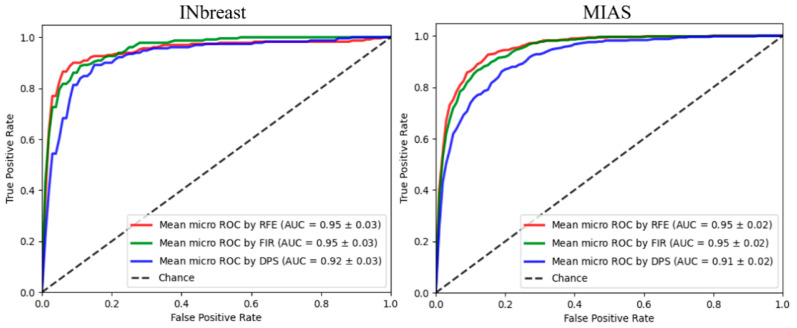
AUCROC values on two datasets using different feature selection methods.

The histogram and K-spectrum features are further concatenated to create a new hybrid feature space. In this step, feature selection procedure becomes more important, as the concatenation operation doubles the feature dimensionality from 656 to 1312. Figure 16 shows the classification results using the hybrid feature vector. A higher CA value 92.76% on INbreast and 86.96% on MIAS are obtained using RFE when *N* = 80 and *N* = 127, respectively, exceeding the best CA using only histogram or K-spectrum features. The AUCROC values are 0.95 ± 0.03 and 0.95 ± 0.02 on two datasets (Figure 17). Since the classification accuracy is improved significantly on both datasets after combining two feature sets, we can conclude that features extracted from K-spectra are complementary to histogram features and further improve the classification accuracy.

### 5.4. Methods Comparison on the INbreast Dataset

In this section, different feature extraction methods are compared for the INbreast dataset. As INbreast has not been used widely with the breast density classification task in the literature, the only available experimental results based on this dataset are 86% and 80.5%, as reported in [25,26], with half images (MLO views) of the dataset tested. We compare five progressive transformations of LBP/LQP methods and implement corresponding algorithms to classify INbreast mammograms, performing comparative analysis based on their classification results. We use RIU4-LQP-K and RIU4-LQP-HK to denote texture feature sets based on the K-spectra and the combination of histograms and K-spectra. Classification performance is evaluated by classification accuracy (CA), AUCROC, Kappa coefficient, and F1 score. In addition, a statistical hypothesis test is conducted. The ‘10-fold cv *t*-test’ method in [43] with a significance level of 0.05 (i.e., *alpha* = 0.05) is employed between RIU4-LQP-HK and every other method to calculate *p*-value, which shows the statistical difference between them. Table 2 shows that our proposed method outperforms other approaches, with the highest CA (91.87 ± 6.28), Kappa (0.89), and F1 score (0.92). In the *t*-test, all other methods present low *p*-values (<0.05), which means the difference in classification performance is statistically significant.

### 5.5. Methods Comparison on MIAS Dataset

Since MIAS has been used in several research works with the breast density classification task, we first compare the five progressive transformations of LBP/LQP methods on it, and then our proposed method is compared with other approaches in the literature. In Table 3, we can see that the proposed RIU4-LQP-HK method provided the best classification performance on the MIAS dataset, with the highest CA (90.61 ± 4.87), AUCROC (94.39 ± 2.88), Kappa (0.86), and F1 score (0.90). In addition, we notice that the classification accuracy obtained can be affected by a few factors, such as the image number used in the test, the results evaluation method, and target categories. Therefore, when comparing our method with other approaches, these factors are considered for presenting an objective and fair comparison. In Table 4, the CA values are compared between different methods, and we can see that the proposed RIU4-LQP-HK method is very competitive with state-of-the-art approaches, providing the highest CA when testing all images in the MIAS dataset.

### 5.6. Effect of Feature Selection

Three feature selection methods are used and compared in all experimental analyses in this paper. In related work [25,44], the DPS method was used to optimise texture features for analysing mammograms and other texture images. However, there was no comparative analysis including other feature selection methods. To bridge this gap, we use the selection results by DPS and corresponding classification accuracy as the base line in this work, and use two other feature selection methods, RFE and FIR, to repeat the feature selection and breast density classification procedures. Comparisons are given in Table 5, from which we can see that RFE works better than the other two methods, with a low number of features used and higher classification accuracy obtained.

### 5.7. Running-Time Comparison

This section compares running-time cost using the proposed classification model. Note that the time cost is recorded for the 10 run 10-fold cross validation in which one fold images are used as the test set and remaining images are used to train the classifier for each iteration. The total time is divided by the number of training and testing images separately in a dataset, and we obtain the average time for processing one image. The methods outlined in this paper are implemented in Matlab R2017b on a desktop computer with Intel core i7 3.6 GHz CPU, 16 GB memory. In Table 6, we can see that the RIU2-LQP- and RIU4-LQP-based methods have similar time cost, requiring around 13~15 ms per image for training. By contrast, the use of LQP costs around 50 times longer time than the other methods on the two datasets due to its high feature dimensionality.

## 6. Discussion

This paper introduces two improvements of the basic LQP method. The first is the RIU4-LQP, which is an extension of the LQP. The second is a novel spatial feature extraction method based on Baddeley’s K-inhom function.

The proposed RIU4-LQP decreases the high feature dimensionality of LQP from 2^P + 2^ to P^2^ + 11, as discussed in Section 3.2. The significantly reduced dimension of the feature space makes it possible to consider a larger neighbourhood of pixels (i.e., higher value of *P*) when computing local texture patterns, while avoiding the exponential rise of the number of features. From the running-time cost shown in Table 6, we can see that the RIU4-LQP-based method only uses around 2% of the time consumed by the basic LQP method. In addition, the proposed RIU4-LQP is rotation invariant, and more texture information related to microstructures can be captured by it as wider spatial transition numbers (*T*) are considered. As discussed in Section 3.3, the texture feature discrimination capability is improved in RIU4-LQP, based on which we assume that a better classification performance for grouping mammogram images can be obtained. The experimental results in Table 2 and Table 3 support this argument: comparing to RIU2-LQP, the classification accuracy is improved by 2% on the INbreast dataset and 8% on the MIAS dataset. Such improvement shows empirical evidence that the proposed RIU4-LQP indeed captures more texture features effectively and helps classify mammograms with higher accuracy.

For the design of a spatial feature extraction method, our main consideration is that every pixel in an image has its own RIU4-LQP code related to the local texture structure. Previous work in this area used the histogram to represent texture features that only gave the frequency of each code and ignored their spatial distribution characteristics. In Section 3.4 and Section 3.5, we address a novel spatial feature extraction framework using Baddeley’s K-inhom function to collect spatial distribution information based on RIU4-LQP. Here, we assume that the extra spatial features provide complementary information about image texture structures that are not obtained from histograms. From experimental results in Section 5.4 and Section 5.5, we can see that the classification accuracy using only RIU4-LQP-based K-spectrum (i.e., RIU4-LQP-K) does not surpass the results by RIU4-LQP-based histograms, with the CA of 84.38% vs. 85.62% on INbreast and 73.33% vs. 80.91% on MIAS. Meanwhile, we observe that the ability of RIU4-LQP-K for the mammogram classification task is similar to RIU2-LQP: 84.38% vs. 83.75% on INbreast and 73.33% vs. 72.73% on MIAS. This indicates that the spatial features extraction using our proposed method performs well, yielding slightly higher performance than RIU2-LQP. Therefore, we further concatenate RIU4-LQP and RIU4-LQP-K to construct a new feature set, RIU4-LQP-HK, which contains both histogram statistics and the spatial distribution features. As shown in Table 2 and Table 3, the improvement on classification performance is significant: taking the RIU4-LQP results as the baseline, CA is improved by 6.25% on INbreast and 9.70% on MIAS. Such improvement is the result of adding the extra spatial features (i.e., RIU4-LQP-K), thus supporting the argument that spatial distribution characteristics can offer some supplementary features that are ignored by histogram statistics. Note that a recent work [18] using a CNN model to classify mammograms reached 88% of accuracy on a large clinical dataset containing 1985 images, but obtained 70% of accuracy on INbreast. The authors in that work argue that the reason comes from the use of a small dataset for training deep CNNs (Figure S2). This also indicates that when training data are limited, our proposed texture feature descriptor is competitive to produce better results, even comparing to neural network models.

Regarding the choice of classification model, various classifiers have been used in the literature, including SVM [8,9,23,25], KNN [6,13], RF [10], Bayesian [21], and MLP [26]. SVM was commonly used and produced higher classification accuracy than others [11,26]. This study therefore uses SVM to process the final feature set, and the grid-search method to select the optimum parameters for it. By contrast, feature selection was not considered and analysed carefully in related work. Some work [8,9,10,11,12,13,23] did not design a feature selection step, instead directly input initial extracted features into a classifier, while others [21,25,26] selected features prior to using a classifier but did not present a comparative analysis. Note that feature selection discussed in this paper refers to the removal of redundant features and compressing feature space for presenting the best classification accuracy. Redundant features that do not closely relate to the target density category can increase the complexity of the classification model and decrease its classifying accuracy. The work reported in this paper particularly gives attention to this point by imposing three feature selection methods and comparing their influences on classification performance.

As shown in Figure 14, Figure 15 and Figure 16, our experimental results provide detailed comparisons when applying different feature selection schemes to two mammogram datasets. From these results, the influence on classification accuracy caused by using different methods is apparent. For example, the CA results on INbreast in Figure 15, the accuracy exceeds 80% when using the top 30 features selected by the RFE method, while it declines to less than 10% after using more than 250 features. This finding also demonstrates that the initial extracted feature space contains some redundant features that do not closely relate to the target class properties (e.g., mammographic density categories in this work). Therefore, removing those redundant features and only keeping the target-related features in the final feature vector is important for producing desirable classification performance. Our analysis on different feature selection methods and feature descriptors are displayed in Table 5, based on which we conclude that for the breast density classification work the RFE performs better than the other two approaches after testing two mammogram datasets. RFE is not used in past work, and we suggest that this can be an important factor to improve the classification results.

As the RFE and other two feature selection methods, work by re-sorting the extracted features to position the most powerful features ahead and redundant features back, it is unclear of how much proportion the spatial features account for in the final selected feature set. We therefore investigate how different features contribute to the final classification results by tracking their orders in the selection procedure. Table 5 shows that there are 80 features in the final feature vector used to obtain the best CA on INbreast, and we find out that 42% of features (i.e., 34 out of 80) contributed by spatial characteristics and the remaining are from histograms: for MIAS, K-spectrum spatial features account for 48% (i.e., 61 out of 127) of the optimised feature set. This gives further evidence that the spatial features extracted by our proposed method create an important and complementary feature set compared to the commonly used histograms, thus improving the capacity of representing image texture features.

Some inconsistent result patterns between the two mammogram datasets are noticed. For example, in Figure 14, while presenting the classification accuracy vs. selected features based on RIU4-LQP histogram features, the RFE method gave the highest CA on INbreast, while the FIR led to the best result on MIAS. Figure 15 shows different CA trends along with the feature number when using RIU4-LQP-K: for INbreast, CA curves saw a significant fall after peak points observed with RFE and FIR methods, while for MIAS, CA curves did not slide too much, but remained comparatively flat until the maximum feature number was used. Such inconsistency between the two datasets can be ascribed to the difference in image types and their classification criteria. As introduced in Section 2, mammograms in INbreast are FFDM images and MIAS is an SFM dataset, with different image qualities. INbreast mammograms are classified based on four BI-RADS density categories, while MIAS uses a three-class (i.e., F-G-D) standard. However, when we test the datasets using the concatenated feature set in Figure 16, a trend in consistent results is seen, with the best performance obtained using the RFE and the worst produced by the DPS.

The sensitivity of machine-learned solutions developed as diagnostic aids must be valid and applicable in clinical practice. The proposed method is based on a novel texture feature descriptor that extracts image microstructure patterns from mammograms. This procedure will not be affected by diagnostic ability of radiologists. However, our method needs a training set with radiologists’ annotations for breast density, based on that a classifier is trained and used to predict the density labels for unseen mammograms. The proposed model was tested on two mammogram datasets that used different density classification criteria and ground truth annotated by different radiologists. Experimental results show that the method maintains good sensitivity on both datasets. Note that the mammogram images in the two datasets were carefully interpreted and annotated by at least two radiologists, and if there was a disagreement, a diagnosis from the third radiologist was required. As such, a comparatively accurate ground-truth set could be built for training the classification model, which guarantees the sensitivity of the method. If the annotations were given by only one radiologist who poses different diagnostic ability, this could lead to some inconsistent density labels compared to other radiologists’ diagnoses and cause a variation in the sensitivity of the classification method.

The proposed method has some distinct advantages. Firstly, the proposed texture feature descriptor is found to be more powerful than the basic LBP/LQP methods in capturing local texture patterns. Secondly, our method, similar to LBP, is easily adaptable to similar applications requiring texture feature extraction. Finally, a novel aspect of this approach is the spatial distribution analysis of texture features, which is important but ignored in past work. Experimental results demonstrate that spatial distribution characteristics provide supplementary image features and effectively improve the classification performance.

In addition to the state-of-the-art classification results obtained, there are some limitations using the proposed methods. The proposed method requires a number of parameters (listed in Table 1) to be optimised first for achieving the best classification results. This is a one-off task which is not repeated in training or testing cycles. In addition, this work was initially inspired by related work of breast density classification, so we continue it with two improvement designs based on the LQP method. Our experimental settings also focus on the mammogram dataset test and its results analysis. We have not tested the effectiveness of the proposed methods on other image datasets. Although we have demonstrated that the proposed RIU4-LQP is rotation invariant and is powerful to capture more microstructures, different image types should be used to test it and give more evidence. We shall address this part of the research in future work.

## 7. Conclusions

This paper presents a robust texture feature descriptor for analysing mammograms and classifying breast density. Based on the conventional LQP operator, the rotation invariant method and different transition number conditions are considered to develop the novel texture descriptor, RIU4-LQP. This paper employs Baddeley’s K-inhom function to capture spatial distribution information of texture feature points, which is used to construct a new feature vector called K-spectrum. After concatenating the histogram and K-spectrum information, this paper also investigates three different feature selection schemes for optimising the initial feature set and improving the classification result. An SVM classifier is trained and used to predict the density labels for test images. Classification results are evaluated by classification accuracy (CA) and AUC, and statistical analysis is conducted between different methods. Two mammogram datasets, INbreast and MIAS, are used to test the proposed methods in our experiments. Experimental results demonstrate that the proposed method extracts robust and effective texture features in mammograms, which improve the classification performance significantly. Comparing with state-of-the-art methods, the classification accuracy by using our proposed approaches is competitive, reaching the best CA of 92.76% and 86.96% on INbreast and MIAS datasets.

## Figures and Tables

**Table 1:** Parameters and their value ranges used in different steps.

Method	Related Parameters
Image pre-processing	1. Resizing scale *s* = 0.125 (on INbreast) and 0.5 (on MIAS) 2. Median filter size: 3 × 3
RIU4-LQP	In multiscale method, three pairs of (*R, P*) are used: 1. (*R*_1_ = 2, *P*_1_ = 10), (*R*_2_ = 4, *P*_2_ = 14), (*R_3_* = 8, *P_3_* = 18) 2. Threshold values {*τ*_1_, *τ*_2_} are decided by an adaptive method
K-inhom function	1. Valid *r*-range: 1~25
Features selection	Initial features number: 1. *n*_1_ = 656 (histogram or K-spectrum features) 2. *n*_2_ = 1312 (concatenated features)
SVM classifier	For grid-searching: 1. Kernel candidates: RBF/Poly/Sigmoid 2. *γ* searching range: [10^−4^, 10^3^] 3. *C* searching range: [10^−3^, 10^4^] 4. *Degree* (only Poly kernel) searching range: {1, 2, 3, 4, 5, 6}

**Table 2:** Classification performance comparison on INbreast using different methods.

	CA (%)	AUC (%)	Kappa	F1	MCC	*p*-Value
RIU2-LBP	78.75 ± 9.35	94.93 ± 1.97	0.71	0.79	0.79	0.0002
RIU2-LQP	83.75 ± 7.50	94.09 ± 2.19	0.78	0.84	0.78	0.0002
RIU4-LQP	85.62 ± 12.20	95.79 ± 2.07	0.80	0.86	0.80	0.0251
RIU4-LQP-K	84.38 ± 8.03	95.37 ± 2.02	0.79	0.85	0.79	0.0028
RIU4-LQP-HK	91.87 ± 6.28	95.36 ± 2.52	0.89	0.92	0.90	-

**Table 3:** Classification performance comparison on MIAS using different methods.

	CA (%)	AUC (%)	Kappa	F1	MCC	*p*-Value
RIU2-LBP	69.38 ± 7.68	84.14 ± 4.54	0.57	0.70	0.57	<10^−4^
RIU2-LQP	72.73 ± 7.55	85.60 ± 4.56	0.59	0.72	0.60	<10^−4^
RIU4-LQP	80.91 ± 8.89	90.72 ± 3.53	0.71	0.81	0.71	0.0099
RIU4-LQP-K	73.33 ± 6.03	87.39 ± 4.15	0.60	0.73	0.60	<10^−4^
RIU4-LQP-HK	90.61 ± 4.78	94.39 ± 2.88	0.86	0.90	0.86	-

**Table 4:** Classification results comparison on the MIAS dataset.

Methods	Image Used	Results Evaluation Method	Results CA (%)	Density Categories
Intensity-based features + SVM [11]	322	Leave-one-image-out	85.7	3 (F, G, D)
Intensity-based features + KNN [11]	322	Leave-one-image-out	78.6	3 (F, G, D)
Intensity-based features + SVM [11]	322	Leave-one-woman-out	77.0	3 (F, G, D)
Intensity-based features + KNN [11]	322	Leave-one-woman-out	76.4	3 (F, G, D)
GLCM + KNN [6]	322	Leave-one-image-out	82.0	3 (F, G, D)
LQP + SVM [25]	322	10-fold cross validation	86.13	4 (BI-RADS)
LBP + Bayesian Network [21]	321	10-fold cross validation	69.4 ± 0.92	3 (F, G, D)
ELBP + Bayesian Network [21]	321	10-fold cross validation	75.4 ± 1.05	3 (F, G, D)
u-ELBP + Bayesian Network [21]	321	10-fold cross validation	73.3 ± 0.64	3 (F, G, D)
M-ELBP + Bayesian Network [21]	321	10-fold cross validation	77.4 ± 1.06	3 (F, G, D)
LDP + Bayesian Network [21]	321	10-fold cross validation	76.0 ± 0.96	3 (F, G, D)
RIU2-LBP + SVM [26]	322	5-fold cross validation	73.8 ± 10.6	4 (BI-RADS)
RIU2-LTP + SVM [26]	322	5-fold cross validation	81.0 ± 9.5	4 (BI-RADS)
RIU2-LQP + SVM [26]	322	5-fold cross validation	82.1 ± 7.1	4 (BI-RADS)
RIU2-LSP + SVM [26]	322	5-fold cross validation	83.3 ± 8.8	4 (BI-RADS)
RIU4-LQP-HK + SVM	322	Leave-one-woman-out	86.96	3 (F, G, D)
RIU4-LQP-HK + SVM	322	Leave-one-image-out	93.21	3 (F, G, D)
RIU4-LQP-HK + SVM	322	10-fold cross validation	90.61 ± 4.78	3 (F, G, D)
RIU4-LQP-HK + SVM	322	5-fold cross validation	86.25 ± 5.24	3 (F, G, D)

**Table 5:** The number (*N*) of features selected by different methods and corresponding CA values.

Dataset	Feature Selection Methods	Histograms-Based Feature Vector		K-Spectra-Based Feature Vector		Combined Texture Features	
		N	Best CA	N	Best CA	N	Best CA
INbreast	RFE	161	88.16	28	82.89	80	92.76
	FIR	164	83.55	47	75.66	473	84.87
	DPS	260	80.92	538	49.34	581	84.21
MIAS	RFE	156	79.19	422	73.60	127	86.96
	FIR	214	81.06	37	71.43	127	86.65
	DPS	152	78.26	263	69.57	109	77.95

**Table 6:** Time cost for training and classifying procedures by using different methods.

Dataset		Average Time for Classifying Images (Milliseconds per Image)					
		LQP	RIU2-LQP	RIU4-LQP	RIU4-LQP-HK + RFE	RIU4-LQP-HK + FIR	RIU4-LQP-HK + DPS
INbreast	Training	715.26	13.96	14.03	13.62	13.93	14.25
	Test	176.71	3.67	3.97	2.90	3.77	4.19
MIAS	Training	717.05	14.85	14.78	14.63	14.23	14.35
	Test	181.34	3.71	3.99	3.56	3.47	3.49

## Data Availability

The MIAS dataset is available at https://www.repository.cam.ac.uk/handle/1810/250394 (accessed on 1 June 2021).

## Supplementary Materials

The following are available online at https://www.mdpi.com/article/10.3390/s22072672/s1, Figure S1: Challenging cases with inaccurate mask images generated and their adjusted mask images based on manual operations. Figure S2: The Network architecture used in [18] (DC: dilated convolutions. CA: channel-wise attention). References [45,46] are cited in the Supplementary Materials.
