# Supplementary material for: Spatial Distribution Analysis of Novel Texture Feature Descriptors for Accurate Breast Density Classification
Source: Sensors (Basel). 2022 Mar 30;22(7):2672. doi: 10.3390/s22072672 (PMC9002800; doi:10.3390/s22072672)
Supplement: Supplementary file 1 [file sensors-22-02672-s001.zip › sensors-1646217-supplementary.pdf]

Supplementary material

1. Introduction of the Pre-processing Stage

A brief introduction of the pre-processing work for segmenting ROI in mammograms is given in Section 3. This supplementary material introduces further details on the methods used in the pre-processing stage.

Image resizing: A scale factor (s) was used to study the variations in classification accuracy with respect to changes in image size in our previous work [33], and experimental results showed that when the INbreast mammogram images were resized to 1/8 of original size, better classification results were obtained. Therefore, in this step, based on the findings in [33], we resized the INbreast mammogram images, from 3328 × 4084 or 2560 × 3328 pixels to 416 × 510 or 320 × 416 pixels respectively. In addition, the image grey level is adjusted from 14bits to 8bits.

Image enhancement and breast region segmentation: The input mammogram is processed using multi-fractal In-Min method [30] to generate the corresponding alpha-image (Figure 4-b), for enhancing the image contrast. The alpha image is divided into 10 clusters by k-means method, and the cluster with the lowest mean is removed as it represents the background. Morphological opening with a disk of radius of five is applied to the image in order to remove any small objects and retain only the largest image segment that represents the breast. An averaging filter is applied to obtain a smoother border of the breast region. Following this step, a breast region mask with pectoral muscle is obtained (Figure 4-d).

Pectoral muscle recognition: Within the breast region segmented using the mask image, intensity based image clustering is performed using the k-means algorithm. For this, the method used in [32] is implemented. Three different numbers of clusters are applied: in the first case, the image is divided into two, in the second it is divided into three, and in the third, it is divided into four clusters. In every image, the cluster with the highest mean value is kept. Finally, the top left corner object (for the left side breast) is assumed to represent the pectoral muscle (Figure 4-e)). Polynomial fitting is used with the sampled points along with the pectoral muscle borderline to obtain a smooth contour (Figure 4-f).

However, the pectoral muscle removal from the breast region area can be a challenging work for some difficult cases. Related works address this problem as a separate research topic [45,46] with different approaches proposed, which are beyond the scope of this paper.

Some mammograms in INbreast dataset present blurred boundary lines between the pectoral muscle area and the breast region, which result in inaccurate mask images. We found around 80 mammogram images (approximately 20%) in INbreast with poorly delineated boundaries, and the corresponding mask images were manually corrected with the help of a breast imaging radiologist. Figure S1 shows some examples of the challenging cases and their mask images before and after using manual operations.

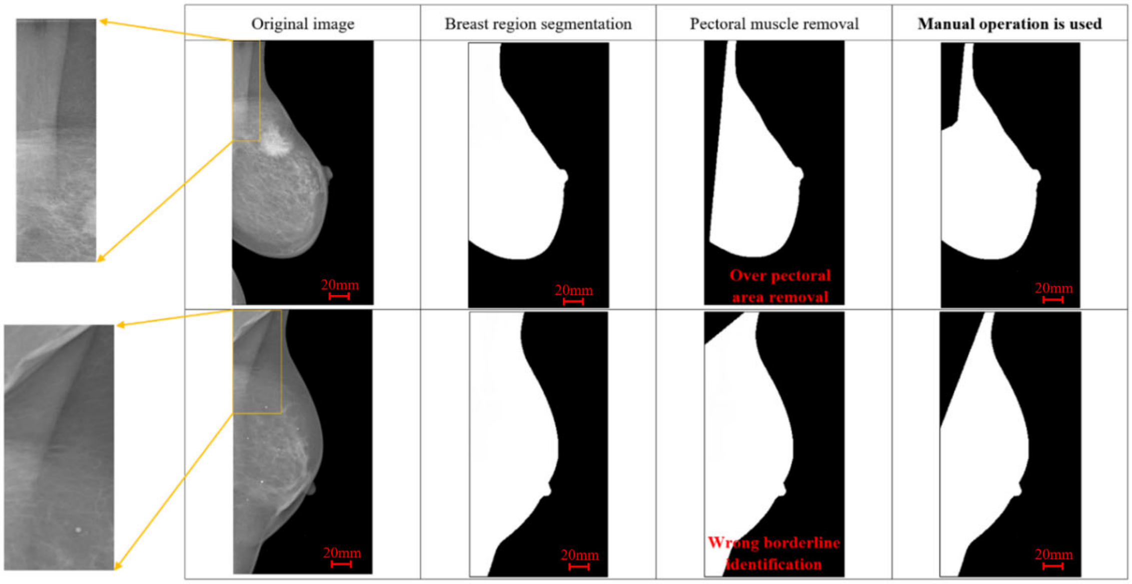

Figure S1. Challenging cases with inaccurate mask images generated and their adjusted mask images based on manual operations.

2. The structure of neural network used in [18] for feature extraction

A recent work [18] proposes a CNN-based radiomics method to extract high-throughput features from mammographs and to classify mammograms into different BI-RADS breast density categories. To improve the classification performance, deep residual learning with dilated convolutions and attention mechanisms is applied on the proposed neural network architecture. The neural network structure used in this work is displayed in Figure S2, where (S1, D1) means that the first convolution in the block has a stride of 1 and all convolutions in the block has a dilation rate of 1. FC refers to fully connected layers.

The proposed method was instantiated with two datasets, one clinical dataset and one publicly available dataset (INbreast), and classification accuracies of 88.7 and 70.0 percent were obtained, respectively.

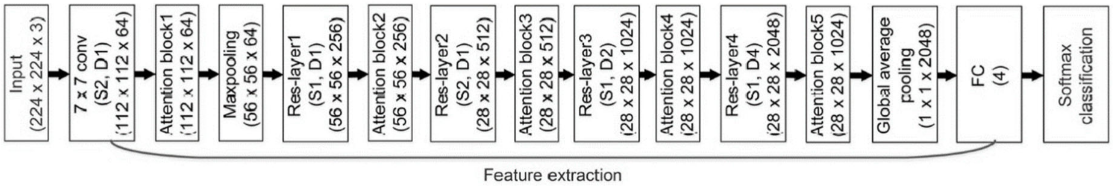

Figure S2. The Network architecture used in [18] (DC: dilated convolutions. CA: channel-wise attention).
